# Therapeutic Potential of Oligo-Fucoidan in Mitigating Peritoneal Dialysis-Associated Fibrosis

**DOI:** 10.3390/md22120529

**Published:** 2024-11-25

**Authors:** Yu-Wei Chen, Mei-Yi Wu, Nai-Jen Huang, Mai-Szu Wu, Yung-Ho Hsu, Chia-Te Liao, Cheng-Hsien Chen

**Affiliations:** 1 Graduate Institute of Clinical Medicine, College of Medicine, Taipei Medical University, Taipei 110, Taiwan; 2 Division of Nephrology, Department of Internal Medicine, Shuang Ho Hospital, Taipei Medical University, New Taipei City 235041, Taiwan; 3 Division of Nephrology, Department of Internal Medicine, School of Medicine, College of Medicine, Taipei Medical University, Taipei 110, Taiwan; 4 TMU Research Center of Urology and Kidney, Taipei Medical University, Taipei 110, Taiwan; 5 Institute of Epidemiology and Preventive Medicine, College of Public Health, National Taiwan University, Taipei 100, Taiwan; 6 Department of Internal Medicine, Division of Nephrology, Wan Fang Hospital, Taipei Medical University, Taipei 116, Taiwan

**Keywords:** fibrosis, mesothelial–mesenchymal transition, MMT, oligo-fucoidan, peritoneal dialysis

## Abstract

Peritoneal dialysis (PD) serves as a home-based kidney replacement therapy with increasing utilization across the globe. However, long-term use of high-glucose-based PD solution incites repeated peritoneal injury and inevitable peritoneal fibrosis, thus compromising treatment efficacy and resulting in ultrafiltration failure eventually. In the present study, we utilized human mesothelial MeT-5A cells for the in vitro experiments and a PD mouse model for in vivo validation to study the pathophysiological mechanisms underneath PD-associated peritoneal fibrosis. High-glucose PD solution (Dianeal 4.25%, Baxter) increased protein expression of mesothelial–mesenchymal transition (MMT) markers, such as N-cadherin and α-SMA in MeT-5A cells, whereas it decreased catalase expression and stimulated the production of reactive oxygen species (ROS). Furthermore, macrophage influx and increased serum pro-inflammatory cytokines, such as IL-1β, MCP-1, and TNF-α, were observed in the PD mouse model. Interestingly, we discovered that oligo-fucoidan, an oligosaccharide extract from brown seaweed, successfully prevented PD-associated peritoneal thickening and fibrosis through antioxidant effect, downregulation of MMT markers, and attenuation of peritoneal and systemic inflammation. Hence, oligo-fucoidan has the potential to be developed into a novel preventive strategy for PD-associated peritoneal fibrosis.

## 1. Introduction

The prevalence of end-stage kidney disease (ESKD) has been increasing globally, resulting in an annual mortality of up to 1.2 million in 2017 [1,2]. The escalating number of patients in need of kidney replacement therapy (KRT) has undoubtedly cast a heavy burden on the healthcare systems in both developing and developed countries [1,3]. Peritoneal dialysis (PD), compared with hemodialysis (HD), is a widely used KRT that provides more patient autonomy and better quality of life [4,5]. From a health-economic perspective, PD is not only more cost-effective but also a sustainable choice, consuming far less water [6,7]. Despite its unique advantages, achieving long-term maintenance of PD therapy remains a challenging issue. One of the major barriers is daily exposure to a large volume of bioincompatible dialysates, which could repeatedly cause peritoneal membrane injury, thus leading to chronic peritoneal inflammation and fibrosis, ending up with irreversible ultrafiltration failure [8,9].

High-glucose dialysate contributes to PD-associated peritoneal fibrosis through multiple mechanisms. Non-physiological high-glucose dialysate with acidity and high osmolarity generates glucose degradation products (GDPs) and advanced glycation end products (AGEs), which all activate TGF-β receptors in mouse mesothelial cells [10,11]. Downstream nuclear translocation of Smad2/3 triggers the transcription of several genes, including Snail, α-SMA, fibronectin, β-catenin, and matrix metalloproteinase-2 (MMP2). This cascade leads to mesothelial–mesenchymal transition (MMT) and ultimately results in peritoneal fibrosis [12,13]. Despite the invention and availability of biocompatible dialysates with physiological acidity and non-absorbable icodextrin dialysate, conventional high-glucose dialysate remains the primary choice in PD prescriptions today [5]. Furthermore, clinical studies have shown that the use of the aforementioned “physiological” dialysates does not avoid the development of pathological peritoneal fibrosis in long-term PD [14].

In addition to MMT, chronic inflammation with macrophage infiltration has been shown to play an important role in peritoneal fibrosis. One study found that depletion of M1 macrophage may reduce the protein expression of α-SMA and fibronectin, thereby preventing MMT and subsequent peritoneal fibrosis. Conversely, the addition of M1 macrophage would induce peritoneal thickening, fibrotic changes, and ultrafiltration failure [15]. Another experimental study also found that total depletion of M2 macrophage by liposomal clodronate could successfully reduce the expression of fibronectin and type I collagen, as well as prevent MMT and the subsequent peritoneal fibrosis [16]. High glucose, GDPs, and AGEs may also trigger heightened oxidative metabolism of glucose and produce reactive oxygen species (ROS). ROS facilitates the activation of proinflammatory cytokines, growth factors, and transcription factors, all of which mediate MMT and cell apoptosis [17]. In human peritoneal mesothelial cells (HPMCs), high-glucose dialysate triggers Beclin 1-dependent autophagy, and inhibiting autophagy ameliorates cell apoptosis, MMT and fibrosis [18]. Despite active research over the past decades yielding a greater understanding of the pathophysiology of PD-associated peritoneal fibrosis, there remains a lack of FDA-approved medications to treat this inevitable complication.

Fucoidan, a sulfated polysaccharide derived from brown seaweed, exhibits multiple pharmacological effects on numerous diseases [19]. Notably, it has shown protective effects against several kidney diseases, such as diabetic nephropathy, chronic kidney disease, and acute kidney injury [20,21,22,23]. The efficacy of fucoidan in treating peritoneal fibrosis among patients undergoing peritoneal dialysis, however, is still uncertain. Fucoidan is recognized for elevating antioxidant levels in serum and tissues, thereby mitigating oxidative stress and cellular damage caused by injury [24,25]. It also possesses anti-inflammatory properties, diminishing endotoxin-induced cellular damage, cytokine release, and neutrophil infiltration [26,27,28]. Additionally, fucoidan inhibits TGF-β1-induced epithelial-to-mesenchymal transition (EMT) in mouse and human renal tubular cells [29,30]. In rodent models of diabetic nephropathy and chronic kidney disease (CKD), oligo-fucoidan significantly decreased interstitial fibrosis in the kidneys [30,31]. These findings suggest that oligo-fucoidan may have the capability to inhibit the development of peritoneal fibrosis.

In this preclinical study, we propose that oligo-fucoidan, a biological macromolecule known for its antioxidative, anti-inflammatory, and antifibrotic effects, could serve as a potential treatment for PD-associated peritoneal fibrosis. To explore its therapeutic potential, we conducted both in vitro and in vivo experiments. We aimed to unravel the underlying mechanisms of action and evaluate the efficacy of fucoidan in mitigating peritoneal fibrosis.

## 2. Results

### 2.1. High-Glucose-Induced Landscape Changes in Protein Expression in Human Mesothelial Cells

**Figure 1:**
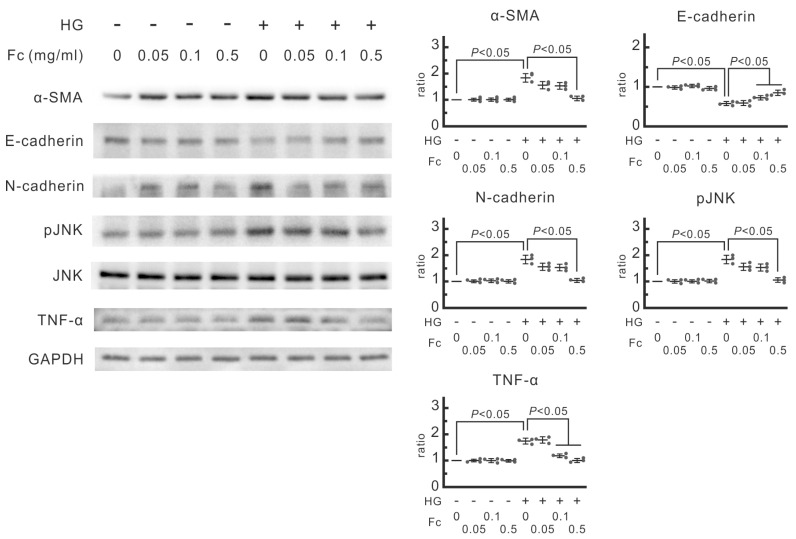
Influence of oligo-fucoidan (Fc) on the signal transduction of epithelial–mesenchymal transition and inflammation in MeT-5A cells. The cells were pretreated with Fc for 30 min and then cultured in high glucose (HG, containing 60 mM glucose) or normal medium. Protein expression was analyzed by Western blotting. α-SMA, E-cadherin, and N-cadherin are the markers of epithelial–mesenchymal transition. Phospho-JNK and TNF-α are the markers of inflammation. The relative increase in protein bands is also presented as a chart. Results are expressed as mean ± SD (*n* = 3).

Supra-physiological high glucose in dialysate causes increased oxidative stress, acute inflammation, and MMT in the peritoneum during PD. To investigate the pathological effects of high glucose, we first analyzed the protein expression in human mesothelial cell MeT-5A treated with or without 60 mM glucose in a medium containing different concentrations of oligo-fucoidan (0, 0.05, 0.1, or 0.5 mg/mL). High glucose led to an increase in the protein expression of α-SMA. Simultaneously, a decrease in E-cadherin and an increase in N-cadherin after high-glucose dialysate (Figure 1, lane 1 and 5). High glucose also induced phosphorylation of JNK and increased TNF-α expression. These data suggested that high-glucose dialysate caused drastic phenotypic changes in MeT-5A cells.

### 2.2. Oligo-Fucoidan Prevents High-Glucose-Induced MeT-5A Cell Apoptosis

**Figure 2:**
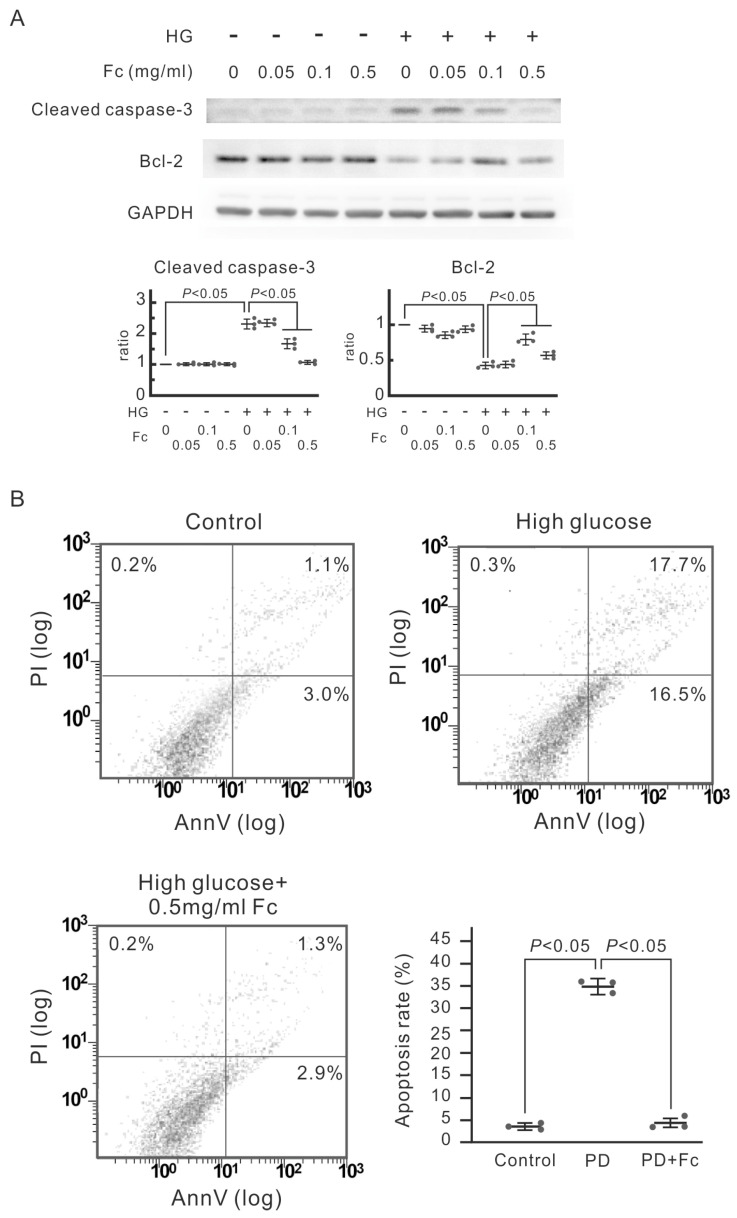
The protective effect of oligo-fucoidan (Fc) on high-glucose-induced apoptosis in MeT-5A cells. The cells were pretreated with Fc for 30 min and then cultured in high glucose (HG, containing 60 mM glucose) or normal medium (control). (**A**) The Western blots of cleaved caspases-3 and Bcl-2. Cleaved caspases-3 and Bcl-2 are the markers of apoptosis. The relative increase in protein bands is also presented as a chart. Results are expressed as mean ± SD (*n* = 3). (**B**) The representative flow cytometric plots of cell apoptosis. In each plot, the lower left quadrant represents viable cells, the upper left quadrant necrotic cells, the lower right quadrant early apoptotic cells, and the upper right quadrant necrotic or late apoptotic cells. The apoptotic rate is also presented as a chart. Results are expressed as mean ± SD (*n* = 3). High-glucose-induced apoptosis in MeT-5A cells, which was inhibited by 0. 5 mg/mL Fc. AnnV: Annexin V-FITC, PI: propidium iodide.

High glucose may cause cell death via glucotoxicity directly or psuedohypoxia and oxidative stress indirectly [32,33,34]. We intended to investigate whether high-glucose-induced apoptosis in human mesothelial cell MeT-5A and whether oligo-fucoidan could prevent high-glucose-induced cell death. Immunoblotting demonstrated that exposure to high-glucose dialysate increased protein expression of the cell apoptosis marker cleaved caspase-3 and decreased expression of antiapoptotic Bcl-2 (Figure 2A). To confirm the apoptosis rate, we utilized flow cytometry to analyze MeT-5A cells incorporated with FITC-annexin V/propidium iodide (PI) double staining. As shown in Figure 2B, MeT-5A cells treated with high-glucose dialysate have a higher apoptosis rate. Oligo-fucoidan prevented the high-glucose-induced expression of cleaved caspase-3 and rescued the downregulation of Bcl-2 (Figure 2A). In addition, pretreatment with oligo-fucoidan also protected MeT-5A cells from high-glucose-induced apoptosis (Figure 2B). Altogether, we substantiated that a higher dose of oligo-fucoidan (0.5 mg/mL) protected human mesothelial cells from high-glucose-induced caspase 3-dependent apoptosis.

### 2.3. Oligo-Fucoidan Inhibits High-Glucose-Induced Oxidative Stress in MeT-5A Cells

**Figure 3:**
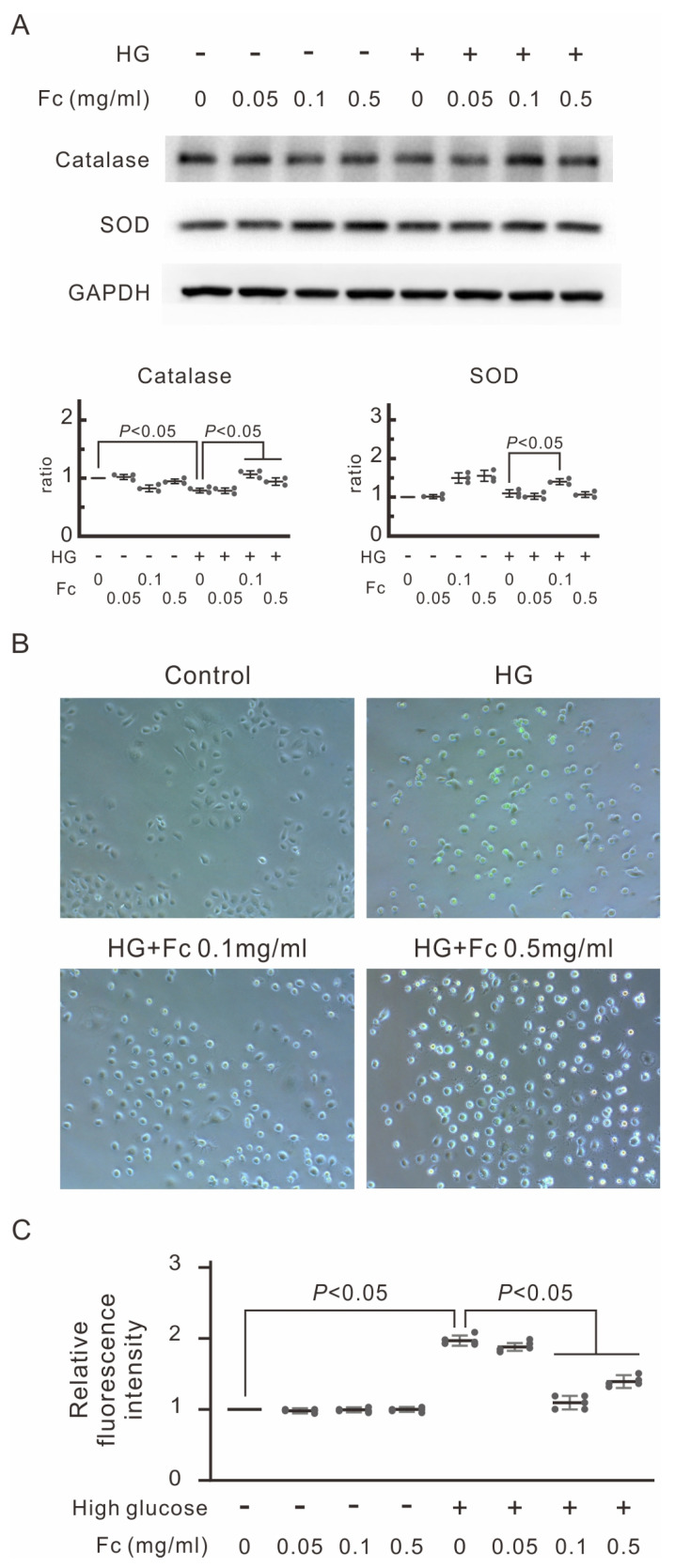
Effects of oligo-fucoidan (Fc) on high-glucose-induced reactive oxygen species (ROS) generation in MeT-5A cells. MeT-5A cells were pretreated with Fc for 30 min and then cultured in high glucose (HG, containing 60 mM glucose) or normal medium. (**A**) The Western blots of catalase and SOD. Catalase and SOD are the markers of antireactive oxygen species. The relative increase in protein bands is also presented as a chart. Results are expressed as mean ± SD (*n* = 3). (**B**) Representative images by fluorescent staining with 2′,7′-dichlorofluorescin (DCF). The image represents a combination of visible light microscopy and fluorescence microscopy images of MeT-5A cells. Green signifies the fluorescence of DCF when stained with ROS. (**C**) The intensity of DCF fluorescence in the cells measured by a fluorescence microplate reader. Fluorescence intensities of cells are shown as the relative intensity of experimental groups compared with untreated control cells. High-glucose-induced ROS in MeT-5A cells, which was inhibited by 0.1 and 0.5 mg/mL Fc. Data are shown in mean ± S.D. (*n* = 5).

The elevated apoptosis observed in MeT-5A cells treated with high glucose could be due to increased oxidative stress. We thus examined the protein expression of antioxidative enzymes in high-glucose-dialysate-treated MeT-5A cells. High-glucose dialysate significantly decreased the expression of antioxidative catalase in MeT-5A cells (Figure 3A). However, adding oligo-fucoidan in the medium prior to high-glucose dialysate could successfully restore the decreased catalase expression. In addition, oligo-fucoidan at a dose of 0.1 mg/mL enhanced superoxide dismutase (SOD) expression compared with MeT-5A cells treated with high-glucose dialysate alone (Figure 3A). To quantify the production of reactive oxygen species (ROS), we incubated MeT-5A cells with a medium containing a fluorescent dye, 2′,7′-dichlorofluorescein (DCF). We then used a spectrophotometer to detect DCF fluorescence intensity as a surrogate of ROS amount. High-glucose dialysate significantly increased DCF fluorescence intensity nearly two-fold, while oligo-fucoidan at doses of 0.1 mg/mL and 0.5 mg/mL inhibited elevation of DCF fluorescence intensity (*p* < 0.05, Figure 3B,C). Altogether, oligo-fucoidan could rescue the downregulation of catalase caused by high glucose. Furthermore, oligo-fucoidan also prevented high-glucose-induced ROS generation.

### 2.4. Oligo-Fucoidan Inhibits PD-Associated Peritoneal Fibrosis and Apoptosis

**Figure 4:**
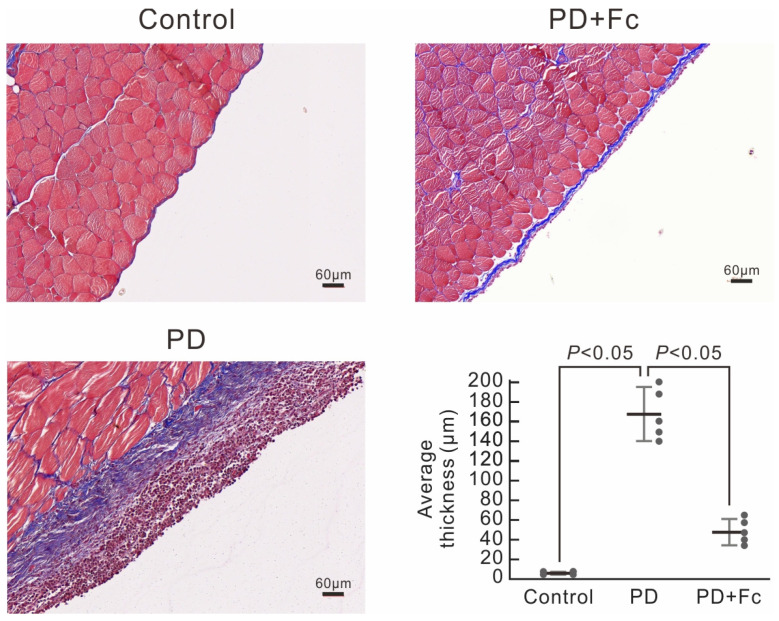
Masson trichrome staining of the parietal peritoneum of PD mice. PD mice received 4.25% dextrose dialysate or without (control) and were then treated with oligo-fucoidan (Fc) orally (100 mg/kg/d). Average parietal peritoneum thickness in PD mice was presented as a chart. Dialysate increased parietal peritoneum thickness in PD mice, which was inhibited by Fc. Data are shown in mean ± S.D. (*n* = 5).

**Figure 5:**
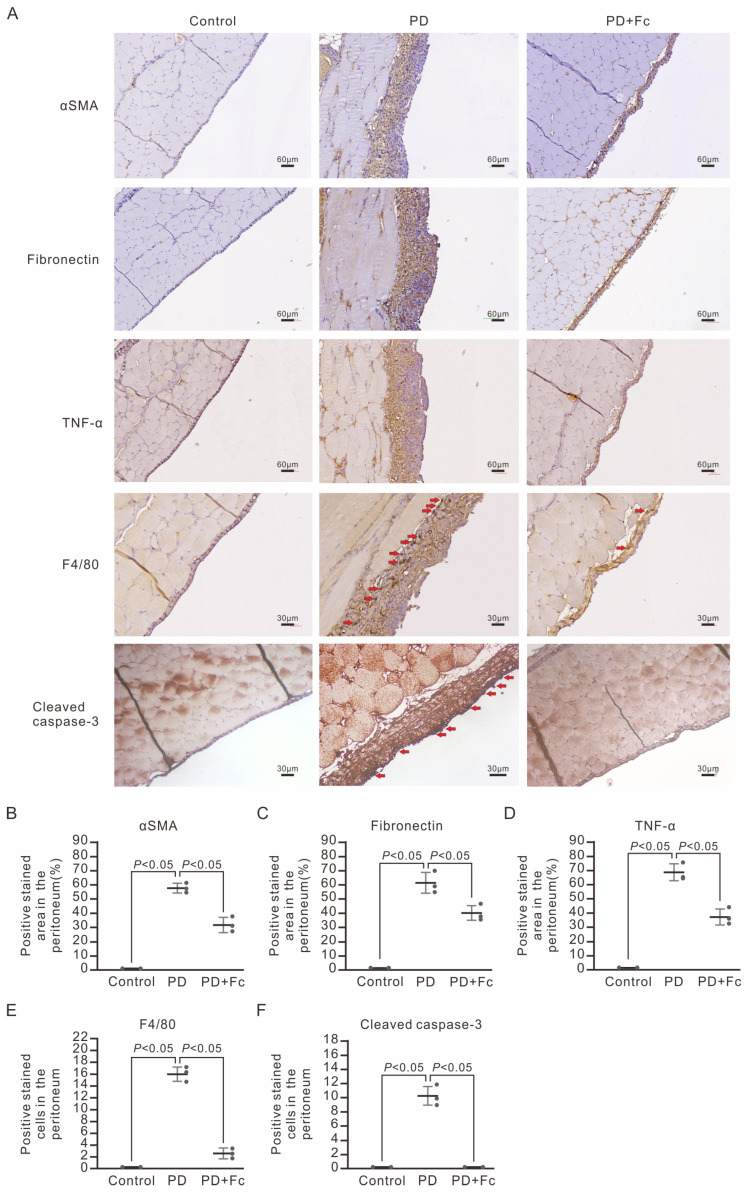
Morphology of the parietal peritoneum of PD mice with or without the oral oligo-fucoidan (Fc) treatment. Sections of paraffin-embedded mouse parietal peritoneum were stained by IHC using antibodies for α-SMA, fibronectin, TNF-α, F4/80, and cleaved caspase-3 (**A**). Red arrows highlight F4/80-positive macrophages and cleaved caspase-3-positive cells in the parietal peritoneum. Charts also display the positively stained areas in the peritoneum for α-SMA (**B**), fibronectin (**C**), and TNF-α (**D**), as well as the positively stained cells for F4/80 (**E**) and cleaved caspase-3 (**F**). Data are presented as the mean ± SD from 3 mice in each group.

We next questioned whether high-glucose dialysate exposure would cause peritoneal fibrosis and thickening and whether oligo-fucoidan has protective effects in vivo. We used a PD mouse model [35] and carefully examined the parietal peritoneum under Masson’s trichrome stain to confirm peritoneal fibrosis. After implantation of the mouse-4-french port, 2 mL of 4.25% dextrose dialysate was injected into the port daily, 5 days per week, for 4 consecutive weeks to mimic peritoneal dialysis (PD). In the control group, a mouse-4-french port with a catheter was implanted, but no dialysate was injected. As shown in Figure 4, 4.25% dextrose dialysate induced severe peritoneal thickening compared with the control group (peritoneum average thickness 167.6 ± 26.1 μm versus 6.4 ± 0.5 μm, *p* < 0.05). We further applied immunohistochemical staining of α-SMA and fibronectin for confirmation of MMT. Peritoneal fibrosis with significantly increased expression of α-SMA and fibronectin was observed in the PD group (Figure 5A–C). Oral gavage of oligo-fucoidan at a dose of 100 mg/kg/d was given concomitantly in the treatment group to validate the benefits of oligo-fucoidan. Notably, enteral administration of oligo-fucoidan substantially prevented peritoneal thickening (Figure 4). The IHC also revealed significantly less staining of α-SMA and fibronectin in the oligo-fucoidan-treated group (Figure 5A–C). Altogether, we found that high-glucose dialysate induced MMT, leading to subsequent peritoneal fibrosis and thickening. And oligo-fucoidan successfully prevented MMT and the development of PD-associated peritoneal pathological changes. Additionally, we observed that cleaved caspase-3 was present in the peritoneum of the PD mouse group, a finding not seen in the control group or the oligo-fucoidan-treated PD group (Figure 5A,F). This suggests that high-glucose dialysate induces apoptosis in peritoneal cells, whereas oligo-fucoidan treatment markedly reduces this effect. These findings are consistent with the results from in vitro experiments (Figure 2).

### 2.5. Oligo-Fucoidan Attenuates PD-Associated Peritoneal Inflammation and Systemic Inflammation

Acute and chronic inflammation plays a crucial driving force in the pathogenesis of PD-associated peritoneal fibrosis. We questioned whether high glucose would induce inflammation in human mesothelial MeT-5A cells. After exposure to high-glucose medium, the protein expression of TNF-α was significantly elevated (Figure 1, lane 5). Although the relative quantity of c-Jun N-terminal kinases (JNK) was not different between the lysates derived from MeT-5A cells with or without high-glucose dialysate, the degree of phosphorylation was higher in the high glucose group (Figure 1, lane 1 and lane 5). This observation pointed out that high glucose may lead to JNK phosphorylation and activation of downstream signaling pathways.

In the mouse PD model, IHC also demonstrated increased TNF-α expression in the parietal peritoneum (Figure 5A,D). To elucidate the role of leukocyte infiltration in the peritoneal inflammation in our PD model, we examine IHC staining of F4/80 as the quantification of macrophage involvement. Along with the peritoneal thickening and fibrotic changes, we also observed prominent F4/80-positive macrophage infiltration within the submesothelial compact zone (Figure 5A,E).

**Figure 6:**
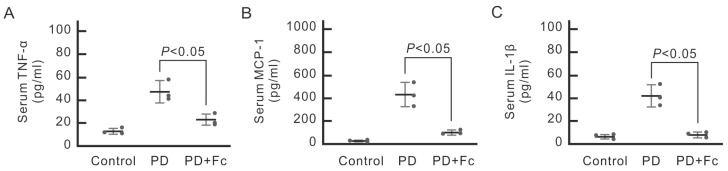
The reduction effect of oligo-fucoidan (Fc) on serum TNF-α, MCP-1, and IL-1β in PD mice. PD mice received 4.25% glucose dialysate or without (control) and were then treated with Fc orally (100 mg/kg/d). Blood was collected from each mouse to measure serum TNF-α (**A**), MCP-1 (**B**), and IL-1β (**C**). Dialysate induced serum TNF-α, MCP-1, and IL-1β in PD mice, which was inhibited by Fc. The results are expressed as the mean ± SD (*n* = 3).

We then questioned whether the irritating high-glucose dialysate would cause not only local inflammation in the peritoneum but also systemic inflammation responses. We thus checked the cytokine levels from PD mouse serum in different groups. The mice that received high-glucose dialysate injections had significantly higher serum concentrations of proinflammatory cytokine TNF-α, IL-1β, and chemokine MCP-1 (Figure 6).

In MeT-5A cells, pretreatment with oligo-fucoidan prevented upregulation of TNF-α and phosphorylation of JNK (Figure 1). Notably, in vivo data also showed that oligo-fucoidan not only inhibits PD-associated local inflammation at the parietal peritoneum but also attenuates systemic inflammation by reducing serum concentrations of pro-inflammatory cytokines (Figure 5 and Figure 6). Altogether, we substantiated that high-glucose dialysate induced TNF-α expression and augmented JNK-dependent downstream signaling, leading to the release of proinflammatory cytokines, whereas oligo-fucoidan counteracted with local and systemic inflammation incited by high glucose.

## 3. Discussion

In the present study, we demonstrated that high-glucose dialysate induces peritoneal thickening and fibrosis in the PD mouse model. These PD-related alterations in the peritoneum may result from several mechanisms: high-glucose-induced mesothelial–mesenchymal transition (MMT), heightened oxidative stress, and subsequent local and systemic inflammation. Furthermore, our findings support the notion that oligo-fucoidan can mitigate oxidative stress, reduce inflammation, and thereby prevent PD-associated peritoneal thickening and fibrosis.

Long-term PD therapy can lead to significant histological changes in the human peritoneum, resulting in altered peritoneal function and eventual ultrafiltration failure [9]. The central role in the pathogenesis of PD-associated peritoneal fibrosis has been attributed to high-glucose-induced MMT mediated by TGF-β1 signaling [36]. High glucose in the dialysate can generate glucose degradation products (GDPs) during heat sterilization. These GDPs contribute to the formation of advanced glycation end products (AGEs) through non-enzymatic reactions. AGEs, in turn, trigger the expression of the receptor for AGE (RAGE). The binding between AGEs in the peritoneal membrane and RAGE on endothelial cells and macrophages activates pro-inflammatory cytokines, perpetuating peritoneal inflammation [32]. High glucose, GDPs, and AGEs all together can activate protein kinase C-α and upregulate downstream type I and type II TGF-β receptors in mouse mesothelial cells [10,11]. The TGF-β1 signaling pathway triggers the phosphorylation of Smad2/3 through type I TGF-β receptors. Subsequent nuclear translocation of Smad2/3 leads to the transcriptional activation of several genes, including Snail, α-SMA, fibronectin, β-catenin, and matrix metalloproteinase-2. The upregulation of these genes ultimately drives mesothelial–mesenchymal transition (MMT) and, eventually, peritoneal fibrosis [12,13,37]. Our investigation revealed that exposure to high-glucose dialysate induced increased expression of α-SMA in human mesothelial cells (Figure 1). Similarly, in a PD mouse model, we consistently observed elevated expression of α-SMA and fibronectin in the mouse peritoneum within the PD group (Figure 5). Notably, we also found that high glucose could induce phosphorylation of JNK in MeT-5A cells, suggesting that the non-Smad-dependent signaling pathway also plays a role in high-glucose-induced peritoneal fibrosis, which aligns with previous studies [32,38,39].

CKD patients are known to suffer from increasing oxidative stress along with disease progression [40]. Additionally, acute peritonitis, whether caused by bacterial or fungal infections, can lead to a significant influx of neutrophils and macrophages. These immune cells produce superoxide through respiratory bursts following cytokine stimulation [17,41]. However, the main culprit of heightened oxidative stress in PD patients is the non-physiological high-glucose conventional dialysate [42]. The substantial influx of glucose into peritoneal cells could exert excessive electron donors through the Krebs cycle [43]. In vitro studies have demonstrated that exposure to commercial PD dialysate (containing 1.5, 2.5, and 4.25% dextrose, which correspond to glucose concentrations of 84 mM, 138 mM, and 236 mM, respectively) results in a 46.3-fold increase in hydrogen peroxide and other ROS production. This exposure also leads to mitochondrial membrane disruption and increased apoptosis in human peritoneal mesothelial cells [44,45]. In our current investigation, we observed that the exposure to 4.25%-dextrose dialysate led to decreased expression of catalase and SOD and nearly doubled overall ROS levels in MeT-5A cells (Figure 3). PD patients may suffer from heightened oxidative stress. From the findings of the current investigation, the downregulation of antioxidative enzymes caused by high glucose may be the underlying mechanism.

Acute peritonitis, often caused by both Gram-positive and Gram-negative bacteria, frequently results in repeated peritoneal injuries and accelerated peritoneal fibrosis, leading to dysfunction. Endotoxin is known to trigger TLR4-dependent chemokine MCP-1 production in murine peritoneal mesothelial cells. LPS exposure in mouse peritoneum initiates the release of several inflammatory cytokines, including IL-1β, IL-6, and TNF-α, leading to further peritoneal fibrosis [46]. Additionally, endotoxin can activate the NLRP3 inflammasome and stimulate IL-1β production in a mouse PD peritonitis model [47]. We observed that high-glucose dialysate alone could prompt TNF-α expression in human mesothelial MeT-5A cells (Figure 1) and increase macrophage infiltration into the peritoneum (Figure 5). Moreover, mice exposed to high-glucose dialysate exhibited significantly higher serum levels of chemokine MCP-1 and inflammatory cytokines, such as IL-1β and TNF-α, compared with control group mice (Figure 6). This study’s findings suggest that high-glucose dialysate exposure causes repeated inflammation on a daily basis. Previous research showed that oligo-fucoidan diminishes hypoxia-induced elevation of MCP-1, IL-1β, and TNF-α in renal tubular epithelial cells. Enteral administration of oligo-fucoidan prevented NGAL expression in an ischemia–reperfusion injury AKI mouse model [48]. Oligo-fucoidan also reduced high-glucose-induced TNF-α expression and JNK phosphorylation in MeT-5A cells (Figure 1), leading to decreased peritoneal macrophage infiltration (Figure 5). In a randomized, double-blind, placebo-controlled trial assessing oligo-fucoidan’s role in asthma patients, it lowered serum IL-1b and IL-6 after 4 weeks and significantly reduced serum IL-8 after 24 weeks [49]. Our in vivo data have also demonstrated that oligo-fucoidan can effectively mitigate systemic inflammatory responses (Figure 6). Consequently, oligo-fucoidan may serve as a novel preventive treatment, shielding patients with PD from recurrent peritoneal and systemic inflammation.

The growing body of evidence underscores the antioxidant potential of fucoidan. It effectively counteracts hydroxyl peroxide and other free radicals [24,50]. As a biological macromolecule, oligo-fucoidan can directly scavenge ROS through redox reaction [24]. Furthermore, we observed that oligo-fucoidan stimulates the expression of catalase and superoxide dismutase (SOD), leading to effective protection from high-glucose-induced ROS formation (Figure 3). Lower oxidative stress associated with oligo-fucoidan use may translate into less local and systemic inflammation, interrupting the vicious cycle of ROS-AGE generation. Moreover, we found that pretreatment of oligo-fucoidan attenuated high-glucose-induced expression of cleaved caspase-3 and mesothelial cell apoptosis (Figure 2). In a mouse CKD model induced by unilateral nephrectomy with ischemia–reperfusion injury to the remaining kidney, oligo-fucoidan alone improved the serum creatinine. Oligo-fucoidan itself prevented hydrogen-peroxide-induced apoptosis as well [21]. Altogether, oligo-fucoidan can reduce high-glucose-dialysate-induced oxidative stress, inhibit apoptosis, and thus preserve the integrity of the peritoneum.

Peritoneal dialysis, as a renal replacement therapy, invariably accompanies CKD or other severe kidney diseases. Peritoneal fibrosis in PD patients is likely also affected by renal disease. The cellular and animal experimental designs in this study did not account for the systemic effects of renal diseases, representing a limitation of this study. However, fucoidan may impact both peritoneal fibrosis and kidney disease concurrently. Research indicates that fucoidan offers protective benefits for several kidney diseases, such as diabetic nephropathy, chronic kidney disease, and acute kidney injury [20,21,22,23]. Additionally, fucoidan has been found to inhibit TGF-β1-induced epithelial–mesenchymal transition in both mouse and human renal tubular cells [29,30]. Oligo-fucoidan has demonstrated a significant reduction in renal interstitial fibrosis in rodent models of diabetic nephropathy and chronic kidney disease [30,31]. The dosage of fucoidan we used in our animal experiments was identical to that used in the above studies. Hence, it can be deduced that PD patients who take fucoidan may experience concurrent mitigation of peritoneal fibrosis and renal diseases. Further clinical trials in the future are required to confirm this inference.

## 4. Materials and Methods

### 4.1. Oligo-Fucoidan

Oligo-fucoidan powder was sourced from Hi-Q Marine Biotech International (Taipei, Taiwan) and produced via enzymatic hydrolysis of the original fucoidan. Briefly, 5 g of fucoidan extracted from *Sargassum hemiphyllum* was mixed in 125 mL of distilled water at 55 °C and stirred, then treated with a glycolytic enzyme at a concentration of 1 mg/g of fucoidan for 6 h [51]. Following centrifugation at 10,000× *g* for 20 min at 4 °C, the supernatant was filtered through a 30 kDa molecular weight cut-off membrane (TangenX Technology Co., Boston, MA, USA), and subsequently, the filtrate was passed through a 1 kDa molecular weight cut-off membrane to yield oligo-fucoidan with an average molecular weight of 800 Da. This oligo-fucoidan, consisting of approximately 4 to 5 L-fucose monosaccharides, was then dissolved in double-distilled water and sterilized with a 0.22 μm filter (Merck Millipore, Darmstadt, Germany).

### 4.2. Cell Culture

We obtained human mesothelial cells MeT-5A from the Bioresource Collection and Research Center (Hsinchu, Taiwan). The MeT-5A cells were cultured in a CO_2_ incubator with the following settings: temperature at 37 °C, humidity at 95%, and 5% CO_2_. We used RPMI 1640 medium supplemented with 15% fetal calf serum (FCS), insulin–transferrin–selenium (ITS), hydrocortisone, L-glutamine, HEPES, and antibiotic/antifungal solution. Prior to the experiments, an overnight culture was performed using a serum-free and hydrocortisone-free medium, as previously documented [52]. To investigate the impact of high glucose on mesothelial cells in vitro, MeT-5A cells were cultured in a medium containing 60 mM glucose, following the methodology outlined in the previous study [53].

### 4.3. The PD Mouse Model

We obtained 12-week-old male 129S1/SvImJ mice from Lasco Technology (Taipei, Taiwan). Male mice were chosen for their consistency in experiments. To create the PD model, mice weighing 21–23 g were initially anesthetized with 5% inhalant isoflurane. Subsequently, a small incision was made on the left flank, and a mouse-4-french silicone port (Access Technologies, Skokie, IL, USA) was implanted. The port was placed at the subcutaneous layer, with the catheter tip inside the peritoneal cavity, following a previously reported protocol [35]. After surgery, the mice were allowed 7 days of post-operative recovery. To mimic human peritoneal dialysis, 2 milliliters of commercial dialysate (4.25%) (product code: FNB9896, Baxter International, Inc., Deerfield, IL, USA) was injected daily via the mouse port. In the control group, mice underwent a sham operation with laparotomy and 4-french silicone port implantation. For the treatment group, oligo-fucoidan was administered to mice through oral gavage at the dose of 100 mg/kg/d. After euthanasia, we dissected the parietal peritoneum of the mid and upper abdomen for histological examination. Under a light microscope, we randomly identified 10 areas of the parietal peritoneum, measured the submesothelial thickness, and calculated the average as the representative severity of peritoneal thickening.

### 4.4. Western Blot Analysis

We loaded MeT-5A cells lysate proteins (15 μg) in each lane for Western blot analysis, as reported previously [31]. We purchased E-cadherin, N-cadherin, p-JNK, JNK, and TNF-α antibodies from Santa Cruz Biotechnology (Dallas, TX, USA); catalase, SOD, and cleaved caspase-3 antibodies from Affinity Biosciences (Cincinnati, OH, USA); Bcl-2 antibody from Cell Signal Technology (Danvers, MA, USA); and GAPDH antibody from Proteintech (Rosemont, IL, USA). After quantifying the protein levels relative to GAPDH, data from five independent experiments were combined and analyzed using Quantiscan software 3.0 (Biosoft, Cambridge, UK).

### 4.5. Immunohistochemical Staining (IHC)

Mouse peritoneal tissues were fixed with 4% buffered paraformaldehyde, embedded in paraffin, and cut into 2 μm sections for staining with UltraVision Quanto Detection System HRP DAB kits (Thermo Scientific, Fremont, CA, USA) and hybridizing with the prime antibody as reported in previous publication [31]. For each mouse, we captured 10 images of the parietal peritoneum at 200× magnification, identified the positively stained areas in the peritoneum, and performed the quantification by software Image-Pro Plus 6 (Media Cybernetics, Rockville, MD, USA) as previously described [31].

### 4.6. Apoptosis Detection

To detect apoptosis induced by high-glucose treatment in MeT-5A cells, we employed FITC-annexin V/propidium iodide (PI) double staining. The MeT-5A cells were harvested and washed twice with ice-cold PBS, and specific binding of FITC-annexin V and staining with PI was performed with an apoptosis detection kit (BD Pharmingen, Franklin Lakes, NJ, USA), according to the manufacturer’s instructions. The cells were then analyzed by BD FACSCanto flow cytometer (BD Pharmingen) to quantify and characterize the apoptotic and necrotic cell populations.

### 4.7. Reactive Oxygen Species (ROS) Detection

We pretreated MeT-5A cells with oligo-fucoidan for 30 min and then cultured them in high-glucose (60 mM glucose) or normal medium for 24 h. To detect ROS, we incubated MeT-5A cells in 96-well plates using a culture medium that included a fluorescent dye, 2′,7′-dichlorofluorescein (DCF), at a concentration of 30 μM for 30 min to stabilize the intracellular level of the probe. The fluorescence intensity of DCF staining within the cells was initially observed with a fluorescence microscope and subsequently quantified using a Varioskan ALF fluorescence microplate reader (Thermo Scientific, Waltham, MA, USA) at excitation and emission wavelengths of 475 nm and 525 nm, respectively. We maintained consistent acquisition parameters and cell numbers across all observations to ensure a valid comparison.

### 4.8. Enzyme-Linked Immunosorbent Assay (ELISA)

The serum samples of the experimental mice were collected and analyzed using TNF-α ELISA kit (R&D Systems, Cat. No. MTA00B), MCP-1 ELISA kit (Abcam, Cat. No. ab208979), and IL-1β ELISA kit (Abcam, Cat. No. ab197742), according to the manufacturer’s instructions.

### 4.9. Statistical Tests

We utilized the Student’s *t*-test and the Mann–Whitney U test to determine the statistical differences between the two groups. Additionally, we conducted a one-way analysis of variance (ANOVA) analysis to compare differences among multiple groups. Differences were defined as significant if *p*-values were less than 0.05. All analyses were completed by the SPSS statistical software V22.0 (IBM Corp., Armonk, NY, USA).

### 4.10. Study Approval

All the animal experiments in the present study were approved by the Taipei Medical University Committee of Experimental Animal Care and Use (approval No. LAC-2019-0513), and we performed animal experiments in compliance with ARRIVE 2.0 guidelines.

## 5. Conclusions

High-glucose Dianeal solution induced the formation of ROS, local and systemic inflammation, and MMT, ultimately leading to peritoneal thickening and fibrosis. Our preclinical study substantiates that oligo-fucoidan, an oligosaccharide extract from brown seaweed, attenuates oxidative stress, rescues mesothelial cells from apoptosis, reduces local macrophage influx, and decreases systemic production of pro-inflammatory cytokines. Considering the promising results observed in mouse models of PD and in vitro experiments using human mesothelial MeT-5A cells, we propose that oligo-fucoidan may serve as a novel therapeutic agent to prevent PD-associated peritoneal fibrosis.

## Data Availability

All data were presented within the article. The original contributions presented in the study are included in the article. Further inquiries can be directed to the corresponding authors.
